# Modulation of the Intestinal Barrier Integrity and Repair by Microbiota Extracellular Vesicles through the Differential Regulation of Trefoil Factor 3 in LS174T Goblet Cells

**DOI:** 10.3390/nu15112437

**Published:** 2023-05-24

**Authors:** Yenifer Olivo-Martínez, Manel Bosch, Josefa Badia, Laura Baldomà

**Affiliations:** 1 Secció de Bioquímica i Biologia Molecular, Departament de Bioquímica i Fisiologia, Facultat de Farmàcia i Ciències de l’Alimentació, Universitat de Barcelona, 08028 Barcelona, Spainjosefabadia@ub.edu (J.B.); 2 Institut de Biomedicina de la Universitat de Barcelona (IBUB), 08028 Barcelona, Spain; 3 Institut de Recerca Sant Joan de Déu (IRSJD), 08950 Barcelona, Spain; 4 Unitat de Microscòpia Òptica Avançada, Centres Científics i Tecnològics, Universitat de Barcelona, 08028 Barcelona, Spain; mbosch@ub.edu

**Keywords:** gut microbiota, postbiotics, extracellular vesicles, inflammatory bowel disease, intestinal barrier, mucosa healing

## Abstract

Trefoil factor 3 (TFF3) plays a key role in the maintenance and repair of intestinal mucosa. TFF3 expression is upregulated by the microbiota through TLR2. At the posttranscriptional level, TFF3 is downregulated by miR-7-5p. Reduced TFF3 levels have been detected in the damaged tissue of IBD patients. Here, we investigate the regulation of TFF3 expression by microbiota extracellular vesicles (EVs) in LS174T goblet cells using RT-qPCR and inhibitors of the TLR2 or PI3K pathways. To evaluate the subsequent impact on epithelial barrier function, conditioned media from control and vesicle-stimulated LS174T cells were used to treat Caco-2 monolayers. The barrier-strengthening effects were evaluated by analysing the expression and subcellular distribution of tight junction proteins, and the repairing effects were assessed using wound-healing assays. The results showed a differential regulation of TFF3 in LS174T via EVs from the probiotic EcN and the commensal ECOR12. EcN EVs activated the TFF3 production through TLR2 and downregulated miR7-5-p through PI3K. Consistently, high levels of secreted TFF3 reinforced the tight junctions and stimulated wound healing in the Caco-2 cells. ECOR12 EVs did not cause these effects. TFF3 is a potential therapeutic target in IBD. This study contributes to understanding the molecular players (microbiota EVs) connecting gut microbes to health and may help in designing better nutritional interventions based on microbiota bioactive compounds.

## 1. Introduction

Intestinal homeostasis and human health greatly depend on the gut microbiota. This complex microbial community contributes to the essential host functions in nutrient and drug metabolism, the development and modulation of the immune system, the maintenance and modulation of mucosal barrier integrity, and protection against pathogen colonization [1]. Microbiota functions are not restricted to the gastrointestinal tract. There is now evidence that the gut microbiota and their metabolites communicate with extraintestinal organs and tissues through several routes [2,3]. Imbalances in the gut microbiota’s composition and function have been associated with a diversity of chronic diseases, including not only intestinal inflammatory disorders, but also autoimmune, metabolic, and neurological illnesses [4]. These findings encourage microbiome-oriented therapeutic approaches to treat dysbiosis-related diseases. In addition to probiotics (alone or as symbiotics), the administration of bioactive compounds produced by probiotics or gut-beneficial bacteria has gained increasing interest in recent years [5,6].

The intestine surface is the primary interface between the microbiota and host. This surface is built from a single layer of epithelial cells covered by a mucus layer, which acts as a physical and chemical barrier to prevent luminal bacteria from reaching the epithelial cells. This layer is composed mostly of mucin glycoproteins (MUC2 in the colon) and contains antimicrobial peptides and IgA secreted by the epithelial and immune cells of the intestinal mucosa [7,8]. Therefore, communication with the host cells principally depends on the microbiota-secreted factors that can pass through the mucus layer. These factors include metabolites, secreted proteins, and extracellular vesicles (EVs) [9]. 

Bacterial EVs are closed, spherical nanostructures derived from the bacterial membrane that allow for the transport and delivery of active bacterial effectors to distal sites in a protected environment provided by the vesicle membrane. They have a great variety of functions that are essential for bacterial survival and the colonization of particular ecological niches [10]. In addition, bacterial EVs are involved in interaction and intercellular communication with the host. Microbiota EVs carry biomolecules produced by the parent bacteria. These include typical microbe-associated molecular patterns (MAMPs) that interact with the immune receptors, known as pattern-recognition receptors (PRRs), of epithelial and immune cells to activate defence and immune responses [11]. Other cargo molecules are proteins, lipids, metabolites, and nucleic acids. Importantly, bacterial EVs can cross the intestinal mucus layer and are internalized by intestinal epithelial cells (IECs) through endocytic pathways. In this way, they deliver their bioactive molecules into the target cells. Some effects of microbiota EVs are strain-specific, since they depend on their cargo and bacterial origin [9]. Today, microbiota-derived EVs are recognized as main players in interkingdom crosstalk. Research efforts in this field have provided scientific evidence that these bacterial EVs mediate microbiota functions by transporting and delivering effector molecules into host cells, which modulate the host’s signalling pathways and cellular processes [9,12]. In this context, we have shown that EVs from probiotic and commensal *Escherichia coli* strains modulate epithelial barrier function [13,14] and activate T cell responses in a strain-specific manner, triggering regulatory events to maintain balanced anti/pro-inflammatory responses [9,15,16]. In addition, oral administration of EVs from the probiotic *E. coli* Nissle 1917 (EcN) ameliorated colitis progression in dextran sodium sulphate-treated mice. At the molecular level, this treatment reversed the altered expression of the pro-inflammatory cytokines and proteins involved in the intestinal barrier function [17]. 

The appropriate physiology and homeostasis of the host are critically associated with the integrity of the epithelial barrier. The disruption of this barrier and subsequent increase in paracellular permeability are important drivers of the pathogenesis of various diseases [18]. Besides the mucus layer, the intestinal epithelium contributes to the gut barrier function by limiting the entry of luminal microbes and harmful compounds. It consists of a monolayer of specialized epithelial cells (enterocytes, goblet cells, M cells, and enterochromaffin cells) sealed by tight junctions (TJs), which control the paracellular passage of molecules, ions, and water. TJs are formed by several types of transmembrane proteins, such as occludin and claudins, and cytosolic zonula occludens (ZO) proteins, which act as scaffolds to anchor the membrane proteins to the actin cytoskeleton [19,20]. In addition, IECs play an essential role in sensing microbial signals through PRRs. Once activated, these cells enhance the intestinal barrier to protect host tissues from bacterial translocation. The gut microbiota modulates the intestinal barrier through several mechanisms that influence TJs, the secretion of antimicrobial peptides from Paneth cells, and the production of MUC2 by goblet cells [21]. Intestinal goblet cells also secrete other proteins that are major components of the intestinal mucus, such as trefoil factor 3 (TFF3) [22].

TFF3 is a small peptide secreted with MUC2 by intestinal goblet cells. It is distributed throughout the mucosal surface of the small and large intestines. TFF3 acts on adjacent epithelial cells in a paracrine manner and plays a key role in the maintenance and repair of intestinal mucosa [23]. TFF3 enhances intestinal barrier function through the regulation of epithelial TJ proteins to reduce paracellular permeability. In this sense, TFF3 upregulates claudin-1 and promotes the redistribution of ZO-1 from the cytoplasm to intercellular junctions [24,25]. TFF3 significantly contributes to mucosa restitution and wound healing by stimulating epithelial cell migration [26,27]. In addition, TFF3 influences mucosal immune responses under the conditions of intestinal inflammation or enteric infections through the regulation of cytokine production [28]. The expression of TFF3 in goblet cells is upregulated in sites of mucosal injury. In addition, the gut microbiota influences the goblet cell function by modulating the expressions of TFF3 and MUC2 through Toll-like receptor 2 (TLR2) signalling [29]. The increased synthesis of TFF3 is followed by the activation of several signalling pathways [30]. The TFF3 effects on cell migration and epithelial integrity preservation during inflammation have been shown to be mediated through the activation of the PI3K/Akt signalling pathway [27,31]. In addition, TFF3 is downregulated at the posttranscriptional level by mir-7-5p [32,33].

TFF3 has been associated with inflammatory bowel disease (IBD) pathogenesis and is considered as a potential treatment target. The expression of TFF3 is downregulated in experimental models of colitis [34] and the damaged tissue of IBD patients, whereas miR-7-5p is overexpressed in IBD samples compared to normal colonic tissue [33]. We showed that, in an experimental model of colitis, the oral administration of EVs from the probiotic EcN increased the TFF3 expression in the colonic tissue to levels similar to those of non-colitic mice [17]. However, the regulatory mechanisms activated by EcN EVs have remained unexplored to date.

This study aims to evaluate the molecular mechanisms involved in the regulation of TFF3 by microbiota EVs in the goblet cell line LS174T. The study includes EVs isolated from the probiotic EcN and the commensal ECOR12. In LS174T cells, the expressions of TFF3 and miR-7-5p were analysed using quantitative RT-PCR in the presence and absence of specific inhibitors of the TLR2 or PI3K signalling pathways. Moreover, to investigate the effect of microbiota EVs on the secreted TFF3 levels and the corresponding impact of the intestinal epithelial barrier, the conditioned medium from the control and vesicle-stimulated LS174T cells was collected and used to treat Caco-2 cell monolayers. In this indirect model, the barrier-strengthening effects were evaluated by analysing the expression and subcellular distribution of selected TJ proteins and the impact on intestinal barrier repair was assessed though wound-healing assays. 

## 2. Materials and Methods

### 2.1. Bacterial Strains and Isolation of EVs

The probiotic *E. coli* Nissle 1917 (EcN) was from Ardeypharm (GmbH, Herdecke, Germany). The *E. coli* strain ECOR12 was isolated from faecal samples of a healthy human adult [35]. 

For the isolation of the EVs, bacterial strains were cultured in Luria–Bertani broth (LB) for 16 h. Then, bacterial cells were pelleted by centrifugation and culture supernatants were used for the extraction of the EVs, as described previously [36]. Briefly, the supernatants were passed through a 0.22 μm pore size filter (Merck, Millipore, MA, USA) to eliminate residual bacterial cells and then concentrated with Centricon Plus-70 centrifugal filters 100 kDa cutoff (Merck, Millipore, MA, USA). After an additional filtration step using a 0.22 μm pore size filter, vesicles were collected by ultracentrifugation at 150,000× *g* for 1 h at 4 °C. The pelleted EVs were washed and resuspended in phosphate-buffered saline (PBS). The quantification of the EV samples was assessed with protein concentration using the Pierce BCA method and sterility was verified on LB plates. Aliquots of the EVs were stored at −20 °C until use.

### 2.2. Cell Culture

Human colonic epithelial Caco-2 cells (ATCC HTB37) were routinely grown in Dulbecco’s modified essential medium (DMEM), supplemented with 10% fetal bovine serum (FBS), 25 mM HEPES, 1% non-essential amino acids, and 1× penicillin-streptomycin (Corning, Product Number 30-002-CI). LS174T human goblet cells were cultured in RPMI 1640 medium, containing 10% FBS and 1× penicillin-streptomycin (Corning, Product Number 30-002-CI). The cultures were maintained at 37 °C in a humidified incubator with 5% CO_2_. The cells were sub-cultured with trypsin-EDTA (Gibco-BRL) once a week.

### 2.3. Stimulation Conditions and Preparation of Conditioned Medium from LS174T Goblet Cells

To analyse the regulation of the TFF3 expression and function via microbiota EVs, two stimulation conditions were set up, depending on the colonic cell line: (i) the direct stimulation of LS174T goblet cells with microbiota EVs, and (ii) the treatment of colonic epithelial Caco-2 cells with goblet-cell-derived conditioned media (GC-CM).

(i) Stimulation of LS174T cells. LS174T cells were seeded at a density of 2 × 10^5^ cells/mL in 60 mm plates or 12-well plates, depending on the experiment, and cultured in RPMI 1640 medium. When the cells reached an 80–90% confluency, the culture medium was replaced by fresh RPMI medium without FBS and the cells were stimulated with EVs (10 µg/mL) isolated from EcN or ECOR12. Non-stimulated cells were cultured in parallel as a control. At the indicated times, the cells were processed for RNA isolation. The culture supernatants were centrifuged at 150,000× *g* for 1 h at 4 °C to obtain the corresponding vesicle-free conditioned media, which were named as EcN-GC-CM, ECOR12-GC-CM, or Control-GC-CM, according to the stimulation conditions. When indicated, LS174T cells were pre-treated for 1 h with the PI3K inhibitor LY294002 (5 µM) or the TLR2 inhibitor C29 (50 µM) before the addition of bacterial EVs. Inhibitors were kept in the culture medium until the end of the experiment.

(ii) Stimulation of Caco-2 cells with GC-CM. Caco-2 cells were seeded at a density of 2 × 10^5^ cells/mL in 6-well plates and cultured in DMEM for 3–4 days to obtain a continuous monolayer. At this point, the cells were washed and treated with the LS174T conditioned media collected from the different stimulations, which were previously mixed with fresh RPMI FBS-free medium (1:1). The cells were incubated for 24 or 48 h, depending on the experiment. 

### 2.4. RNA Extraction and Quantitative Reverse Transcription-Polymerase Chain Reaction (RT-qPCR)

Gene expression was analysed using RT-qPCR in LS174T cells at 6 h and in Caco-2 cells at 24 h post-treatment with the bacterial EVs or GC-CMs, respectively. The total RNA was extracted using the miRNeasy mini Kit (Qiagen, Crawley, UK) according to the manufacturer’s protocol. The RNA quality and concentration were assessed by the ratio of absorbance at 260/280 nm using a NanoDrop TM-2000 spectrophotometer. All the samples had 260/280 ratio values between 1.9–2.0.

For the RT-qPCR, the RNA (1 µg) was reverse transcribed in a final volume of 20 µL using the High Capacity cDNA Reverse Transcription kit (Applied Biosystems, Foster City, CA, USA). Quantitative PCR reactions were performed in a Quantstudio^TM^ 3 Real-Time PCR system (Applied Biosystems) by using SYBR^®^ Green PCR Master Mix (Applied Biosystems) and specific primers for TLR2, TFF3, and MUC2 (for LS174T cells), and occludin, claudin-1, claudin-2, and ZO-1 (for Caco-2 cells) (Table 1). The housekeeping GAPDH gene was used as the internal control gene. The reaction program consisted of one denaturation cycle for 10 min at 95 °C, followed by 40 cycles of 15 s at 95 °C and 1 min at 60 °C. A control reaction was carried out in the absence of RNA.

For the miRNA-7-5p expression analysis, reverse transcription of RNA (5 ng/μL) was performed using the miRCURY LNA RT kit (Qiagen), followed by quantitative PCR using the miRCURY LNA PCR Assay (Qiagen). The U6 snRNA reference gene was used for normalization. The primers used were the hsa-miR-7-5-pMiRCURY LNA primer (Qiagen) (MiRBase accession # MIMAT0000252) and the U6 snRNA control primer set (Qiagen). The standard PCR program consisted of one denaturation cycle for 2 min at 95 °C, followed by 40 cycles of 10 s at 95 °C and 1 min at 56 °C. 

For both the mRNA and miRNA analyses, the relative gene expression was calculated using the 2^−ΔΔCt^ formula. The data were presented as the fold change in gene expression relative to the untreated control [37].

**Table 1:** Primers used in this study for mRNA expression analysis using RT-qPCR.

Gene	Sequence (5′-3′)	Reference
GAPDH	Fw: GAG TCA ACG GAT TTG GTC GT Rv: GAC AAG CTT CCC GTT CTC AG	[38]
TFF3	Fw: CAGCTTTTCTGTCCCTTTGC Rv: CACGACGCAGAAATAAA	[38]
TLR2	Fw: CCGTGGAATGTTTGGAACTGC Rv: ATGCAGCCTCCGGATTGTTA	[31]
MUC2	Fw: CACCTGTGCCCTGGAAGGC Rv: CGGTCACGTGGGGCAGGTTC	[39]
ZO-1	Fw: TGAGGCAGCTCACATAATGC Rv: GGTCTCTGCTGGCTTGTTTC	[38]
Occludin	Fw: TTTGTGGGACAAGGAACACA Rv: TCATTCACTTTGCCATTGGAT	[38]
Claudin-1	Fw: CGATGAGGTGCAGAAGATGA Rv: CCAGTGAAGAGAGAATGACC	[38]
Claudin-2	Fw: ACCTGCTACCGCCACTCTGT Rv: CTCCCTGGCCTGCATTATCTC	[40]

Fw: Primer forward. Rv: Primer reverse.

### 2.5. Quantification of TFF3 by ELISA

LS174T cells, grown on 60 mm plates until they reached an 80–90% confluency, were incubated in the absence or presence of EcN or ECOR12 EVs for 24 h. Culture supernatants were collected, centrifuged at 16,000× *g* for 20 min at 4 °C, and stored at −80 °C until use. The TFF3 secreted levels were quantified by the solid-phase sandwich ELISA kit (Invitrogen, USA, catalogue # EHTFF3), according to the manufacturer’s instructions. The results were expressed as ng/mL.

### 2.6. Confocal Immunofluorescence Microscopy

Caco-2 cells were grown for six days in an 8-well chamber slide (µ-Slide 8 well Glass bottom, Ibidi) and then stimulated with GC-CM (1:1) for 24 h. After being washed with PBS, the cells were fixed with 4% paraformaldehyde in PBS, permeabilized with 0.05% saponin (Sigma-Aldrich, Chemical Co., St. Louis, MO, USA) and blocked using PBS containing 1% bovine serum albumin. The TJ protein ZO-1 was stained using anti-ZO-1 (5 µg/mL, Invitrogen) for 16 h at 4 °C, followed by incubation with Alexa Fluor 488-conjugated F(ab’)2 goat anti-mouse IgG (H+L) (5 µg/mL, Invitrogen, Waltham, MA, USA) for 2 h at 37 °C. Nuclei were labelled with DAPI (0.125 µg/mL, Sigma Aldrich, Chemical Co., St. Louis, MO, USA) for 20 min at room temperature.

For the confocal microscopy, the cells were observed using a Zeiss LSM880 confocal microscope equipped with a 63×/1.4 objective, an Argon laser, and a 405 nm laser diode. The 488 nm line of the Argon laser was used to collect the fluorescence of the Alexa Fluor 488 and the 405 nm diode was used to detect the DAPI emission fluorescence. Images were acquired with a voxel size of 0.13 µm × 0.13 µm × 0.37 µm (x, y, z, respectively). For each experimental condition and replicate (*n* = 3), 5 different fields of view with 30–40 cells each were acquired.

For a quantitative analysis of the TJ intensity, images were processed using Fiji software v1.53t [41], following the protocol described previously [14]. Mainly, they were filtered and processed to remove the background to finally trace the tight junction fluorescence using the Tubeness plugin and project with maximum intensity. Segmentation was performed by using a pretrained Labkit classifier [42]

### 2.7. Cell Viability and Proliferation Assays

Cell viability was assessed using the MTT (3-(4,5-Dimethylthiazol-2-yl)-2,5-diphenyl tetrazolium bromide) assay. For this, 100 µL of a cell suspension of 1 × 10^5^ cells/mL was seeded in a 96-well plate and incubated for 24 h at 37 °C in the appropriate complete medium before treatment. The cells were then challenged with the selected stimuli and incubated for up to 48 h. Specifically, LS174T cells were treated with EcN or ECOR12 EVs (10 µg/mL) and Caco-2 cells were treated with the corresponding GC-CM (1:1), as described above. At 24 h intervals, the viable proliferating cells were measured using the MTT assay, as described previously [43]. This assay was also used to evaluate the cytotoxicity of LY294002 and C29 on the goblet cell line LS174T at 6 h of incubation, with different inhibitor concentrations ranging from 5 to 50 µM. The results were expressed as the percentages of cell survival relative to the controls (untreated cells). 

When indicated, the trypan blue exclusion test was used to assess the cell viability and proliferation. In this case, the cells were plated into 24-well plates, exposed to bacterial EVs (10 µg/mL), and further incubated up to 48 h. At the indicated times, the cells were detached using trypsinization, stained with 0.25% *w*/*v* trypan blue, and counted with a haemocytometer. The Mean Proliferative Index (MPI) at any given point was calculated as previously described [43]. MPI = (number of cells treated with EVs in each well/number of control cells in each well) × 100. 

### 2.8. Wound-Healing Assay

The wound-healing assay was used to evaluate the ability of the microbiota EVs to promote intestinal mucosal healing. Caco-2 cells (2 × 10^5^ cells/well) were grown in a complete culture medium in a 6-well plate equipped with wound-healing assay µ-Dish 25 mm insets (ibidi 80206). When the cells reached confluency (3–4 days), the insets used to produce the scratched area were removed. The wounded cell monolayers were washed with FBS-free RPMI medium and then incubated with GC-CM mixed with FBS-free RPMI medium (1:1). Images were captured under the microscope at 0, 24, and 48 h post incubation (ZOETM Fluorescent Cell Imager-1). The wound closure analysis was performed with the ImageJ v1.53t by measuring the width along the cell-free gap. For each treatment, the results were expressed as the percentages of wound closure at 24 and 48 h compared to the wound at the time of 0 h.

The same assay was used to evaluate the ability of the microbiota EVs to stimulate the migration of LS174T cells. In this case, LS174T cells (2 × 10^5^ cells/well) were grown using the same plates and insets for 2 days. After removing the insets, the cells were washed and incubated in FBS-free RPMI medium in the absence or presence of EcN or ECOR12 EVs (10 µg/mL). A wound healing analysis was carried out at 0, 24, and 48 h.

### 2.9. Statistical Analysis

The data were collected from at least three independent biological experiments in triplicate. GraphPad Prism 7.0 software (GraphPad Software, Inc., La Jolla, CA, USA) was used to perform the statistical analysis and generate graphs. The data for all the measurements are presented as the mean ± standard error (SEM). Differences between the groups were assessed using a one-way analysis of variance (ANOVA), followed by Tukey’s post-test. Significant differences were established at a *p*-value ≤ 0.05. 

## 3. Results

### 3.1. Modulation of TFF3-Related Genes Using EcN and ECOR12 EVs in LS174T Cells

To better understand the molecular mechanisms involved in the regulation of TFF3 via microbiota EVs, the expressions of TFF3 and its regulators, TLR2 and miR7-5p, were studied in LS174T cells, a well-characterized model of human goblet cells [39]. The expression of MUC2, a TFF3-related protein that is co-secreted and synergizes with TFF3 to preserve the integrity of the intestinal barrier, was also analysed.

**Figure 1:**
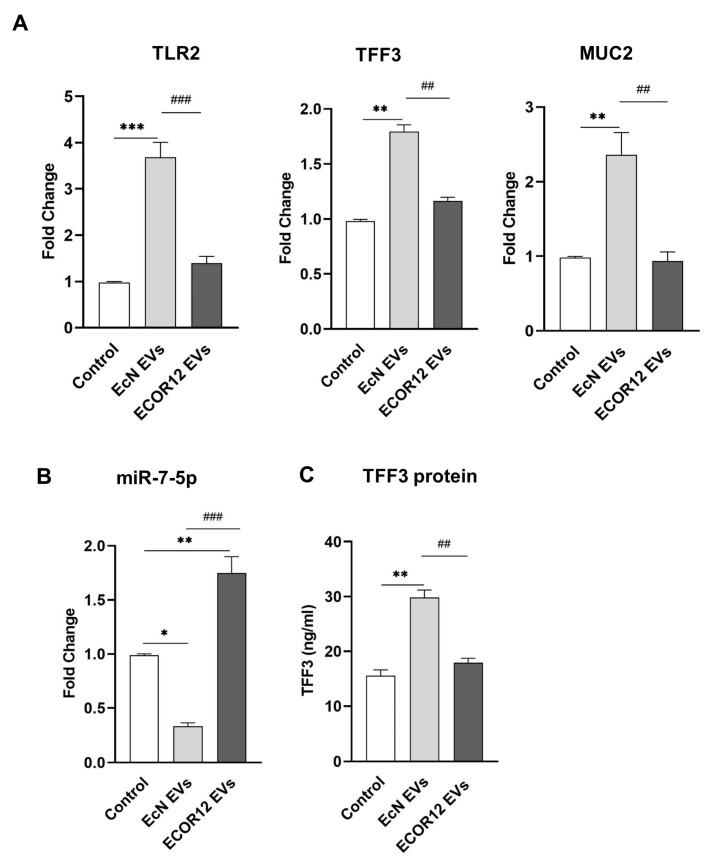
Modulation of TFF3 expression in LS174T cells by microbiota EVs. LS174T cells were challenged with EVs (10 µg/mL) from EcN or ECOR12. Untreated LS174T cells were processed as controls. (**A**) Relative mRNA levels of TFF3, MUC2, and TLR2 were measured using RT-qPCR after 6 h incubation, using GAPDH as the reference gene. (**B**) RT-qPCR quantification of miR-7-5p levels at 6 h post-incubation using the U6 reference gene. In both panels (**A**,**B**), data are presented as fold-change compared to untreated control cells. (**C**) Secreted TFF3 protein was quantified by ELISA after 24 h incubation. Values were expressed as ng/mL. In all panels, data are expressed as mean ± SEM from three independent experiments. Differences were evaluated with one-way ANOVA, followed by post hoc Tukey’s. * *p* ≤ 0.05, ** *p* ≤ 0.01, *** *p* ≤ 0.001 vs. control group, and ## *p* < 0.01, ### *p* ≤ 0.001 between vesicle treatments.

LS174T cells were stimulated for 6 h with EVs (10 µg/mL) isolated from the probiotic EcN or the commensal ECOR12. Untreated LS174T cells were kept as controls. RT-qPCR analysis revealed that the EcN EVs significantly increased the TFF3 (*p* ≤ 0.01), MUC2 (*p* < 0.01), and TLR2 (*p* < 0.001) mRNA levels, whereas the ECOR12 EVs did not cause a significant effect on their expressions, yielding mRNA levels similar to those of the control cells (Figure 1A). 

Regarding miR7-5p, the results showed differential regulation by EcN and ECOR12 EVs (Figure 1B). The treatment with EVs from the probiotic EcN caused a downregulation of the miR7-5p levels (*p* < 0.05) in LS174T cells. In contrast, the miR7-5p levels were significantly increased in the cells stimulated with the ECOR12 EVs (*p* < 0.001) (Figure 1B). The expression of TFF3 was also analysed at the protein level. The secreted TFF3 peptide was quantified using ELISA in LS174T culture supernatants, following a 24 h stimulation with EcN or ECOR12 EVs (Figure 1C). Consistent with the mRNA level profile, the secretion of TFF3 was significantly increased in LS174T cells incubated with EcN EVs when compared to both the control and ECOR12-treated cells (*p* ≤ 0.01). 

The positive correlation between the TFF3 and TLR2 expression levels in LS174T cells treated with EcN EVs suggested that the activation of TFF3 by EVs from this probiotic involves the TLR2 signalling pathway. In these cells, the downregulation of miR7-5p is consistent with the activation of the PI3K/Akt signalling pathway by TFF3 [32]. Concerning ECOR12 EVs, the results obtained suggest that EVs from this commensal can regulate miR7-5p using a different mechanism. 

### 3.2. Effect of TLR2 and PI3K Inhibitors on the Modulation of TFF3 and miR-7-5p by EcN or ECOR12 EVs

**Figure 2:**
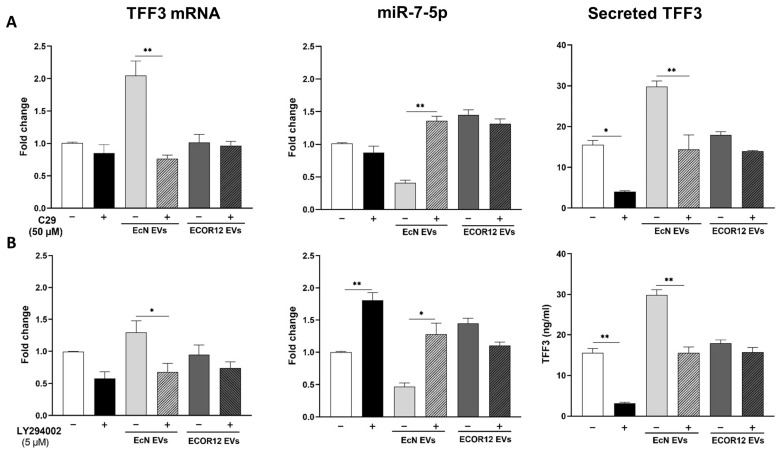
Effect of TLR2 and PI3K inhibitors on TFF3 and miR7-5p expressions in LS174T cells stimulated with EcN or ECOR12 EVs. LS174T cells were pre-treated for 1 h with: (**A**) C29 (50 µM), or (**B**) LY294002 (50 µM) before the addition of EVs (10 µg/mL) from EcN or ECOR12. Inhibitors were kept in the culture until the end of the experiment. Untreated LS174T cells were processed as controls. Relative mRNA levels of TFF3 and miR7-5p were measured using RT-qPCR after 6 h incubation, using GAPDH or U6 as the reference gene, respectively. Data are presented as fold-change compared to untreated control cells. For all treatments, secreted TFF3 protein was quantified by ELISA after 24 h incubation and values expressed as ng/mL ((**A**,**B**), right panels). In all panels, data are expressed as mean ± SEM from three independent experiments. Differences were evaluated with one-way ANOVA, followed by post hoc Tukey’s. For each treatment group (untreated control, treated with EcN EVs, or treated with ECOR12 EVs), * *p* ≤ 0.05, ** *p* ≤ 0.01 indicate the level of statistical significance between cells stimulated in the absence (−) and presence (+) of inhibitor.

To investigate the involvement of the TLR2 signalling pathway and the subsequent activation of PI3K in TFF3 expression induced by microbiota EVs, LS174T cells were pre-treated with the specific TLR2 inhibitor C29 (50 µM) or the PI3K inhibitor LY294002 (5 µM) before the addition of bacterial EVs. For both inhibitors, the concentration was selected based on the cell viability results from a dose–response curve using the MTT assay (Supplemental Figure S1). The TFF3 and miR7-5p expression levels were quantified using RT-qPCR on LS174T cells following a 6 h incubation with EcN or ECOR12 EVs, in the presence and absence of the inhibitors. The non-stimulated control cells were also treated with the inhibitors as a control (Figure 2). The C29-mediated inhibition of the TLR2 signalling pathway significantly abolished the induction of TFF3 by EcN EVs (*p* ≤ 0.01). In addition, the C29 treatment prevented the downregulation of miR7-5p by EcN EVs, reaching expression levels that were even higher than those of the control cells (Figure 2A). In contrast, the expressions of TFF3 and miR7-5p remained unchanged in the ECOR12 EVs-treated cells in the presence of C29 (Figure 2A).

In the non-stimulated control cells, the inhibition of the PI3K pathway with the LY294002 treatment significantly increased the miR7-5p expression levels (*p* ≤ 0.01). Consequently, the expression levels of the target TFF3 mRNA were diminished by this treatment (Figure 2B). The same expression pattern, the upregulation of miR7-5p (*p* ≤ 0.05) and the downregulation of TFF3 (*p* ≤ 0.05), was observed in LS174T cells stimulated with EcN EVs in the presence of LY294002 (Figure 2B). In the cells challenged with ECOR12 EVs, the results indicate that, after the LY294002 treatment, there were no statistically significant changes affecting the TFF3 or miR-7-5p expressions (Figure 2B). 

For both inhibitor assays, the TFF3 secreted levels were quantified in the culture supernatants (Figure 2, right panels). The results were consistent with the TFF3/miR7-5p expression profile, which confirmed their differential regulation by EcN and ECOR12 EVs. The increased secretion of the TFF3 peptide promoted by EcN EVs was significantly reduced (*p* < 0.01) in the presence of either C29 or LY294002, yielding values that were similar to the non-stimulated control cells. In contrast, none of the inhibitors altered the basal TFF3 secretion level in the cells stimulated with the ECOR12 EVs.

Overall, the results evidenced that EcN EVs induce TFF3 expression in LS174T through the TLR2 signalling pathway and downregulate miR7-5p via the subsequent TFF3 activation of PI3K. Contrary to the probiotic EcN, vesicles from the commensal ECOR12 did not induce TFF3 expression in LS174T cells. Moreover, the ECOR12 EVs promoted the upregulation of miR7-5p through a mechanism that did not involve the TLR2 or PI3K pathways.

### 3.3. EcN and ECOR12 EVs Stimulate Restitution of LS174T Cells upon Epithelial Injury

**Figure 3:**
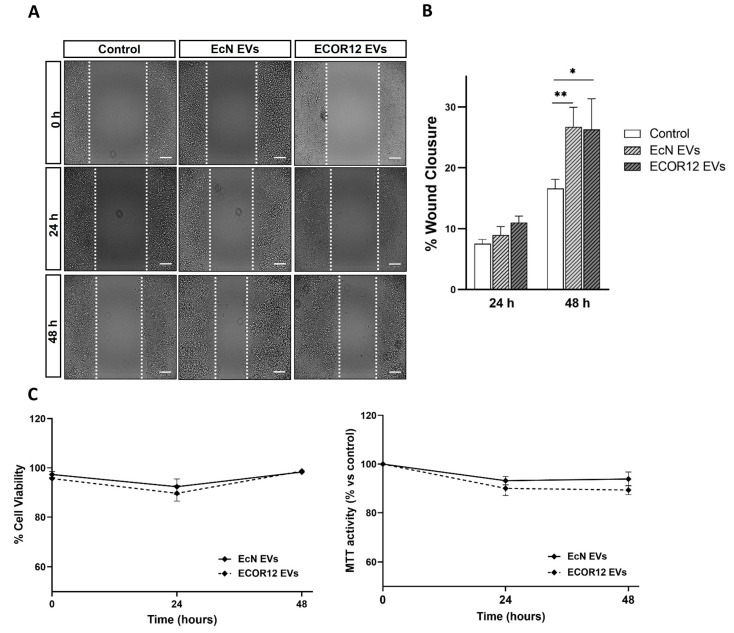
EVs from EcN and ECOR12 activate goblet cell restitution. Wounds were generated in LS174T cells using the culture inset system. After removing the insets, cells were incubated in FBS-free RPMI medium in the presence of EVs (10 µg/mL) from EcN or ECOR12 or without any stimuli. (**A**) Representative images of scratched areas at 0, 24, and 48 h are shown (Scale bar = 100 µm). (**B**) Analysis of wound closure. ImageJ was used to measure the width along the cell-free gap. Results are presented as the percentage of wound closure at 24 h and 48 h compared to the wound at time 0 h and expressed as mean ± SEM. (**C**) Cell viability under the experimental conditions assessed with the MTT and trypan blue exclusion assays. In all panels, data are expressed as mean ± SEM from three independent experiments. Differences were evaluated with one-way ANOVA, followed by post hoc Tukey’s. * *p* ≤ 0.05, ** *p* ≤ 0.01 vs. control group.

Mucosal wound healing involves epithelial cell migration as a first step, followed by epithelial cell proliferation. In this context, the effects of the EcN and ECOR12 EVs on goblet cell migration were assessed using the wound-healing assay. After the culture insets had been removed to create the scratched area, LS174T cells were incubated in FBS-free RPMI medium in the absence or presence of the EcN or ECOR12 EVs, as described in the Material and Methods section. Cell proliferation is significantly inhibited in the absence of FBS. At 48 h, the EVs from both strains had reduced the wound area (26.7 ± 26.3% of closure) in comparison to the control cells (16.6% of closure), without significant differences between the strains (Figure 3A,B).

Under these experimental conditions, the cell viability was confirmed by the MTT and trypan blue exclusion assays (Figure 3C). To evaluate the vesicle effects on cell proliferation, the LS174T cells were incubated in 5% FBS-RPMI in the absence and presence of EcN or ECOR12 EVs for up to 48 h. The cell proliferation was analysed using the MTT assay. The MTT activity values of the vesicle-stimulated cells did not significantly differ from those of the untreated control cells (Supplemental Figure S2).

### 3.4. Effect of the Conditioned Media from EV-Stimulated LS174T Cells on the Expression of TJ Proteins in Caco-2 Cells

**Figure 4:**
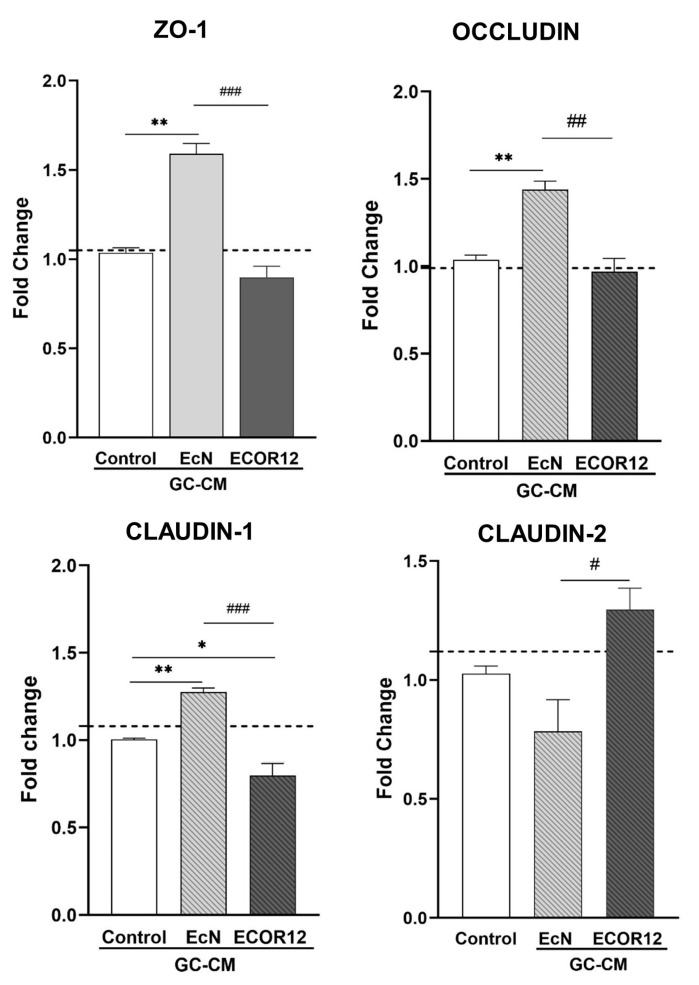
mRNA expression levels of ZO-1, occludin, claudin-1, and claudin-2 in Caco-2 cells treated with EV-stimulated LS174T conditioned media. Caco-2 cell monolayers were treated with GC-CM (1:1) collected from LS174T cells following stimulation with EcN EVs, ECOR12 EVs, or without any stimuli. As a control of GC-CM treatment, cells were incubated in parallel with RPMI (untreated, dashed line). Relative mRNA levels of the indicated TJ proteins were measured using RT-qPCR after 24 h incubation, using GAPDH as the reference gene. In all panels, data are expressed as mean ± SEM from three independent experiments. Differences were evaluated with one-way ANOVA, followed by post hoc Tukey’s. * *p* ≤ 0.05, ** *p* ≤ 0.01 vs. control group, and # *p* ≤ 0.05, ## *p* < 0.01, ### *p* ≤ 0.001, between EcN-GC-CM and ECOR12-GC-CM treatments.

TFF3 plays a key role in the maintenance of the intestinal mucosa. This peptide enhances the integrity of the mucosal barrier by influencing the expression of TJ proteins in intestinal epithelial cells. In this context, we sought to analyse whether the upregulation of TFF3 with EcN EVs in LS174T cells provides an indirect mechanism to reinforce intestinal epithelial TJs. To this end, Caco-2 cell monolayers were treated with LS174T conditioned media (GC-CM) collected from non-stimulated control cells or cells stimulated with EcN or ECOR12 EVs, and the mRNA expression level of major TJ proteins was quantified using RT-qPCR at 24 h of incubation (Figure 4). Caco-2 cell monolayers were incubated in parallel with fresh RPMI (untreated) as a control to discard negative GC-CM effects. 

The treatment with EcN-GC-CM significantly upregulated ZO-1 (*p* ≤ 0.01), occludin (*p* ≤ 0.01), and claudin-1 (*p* ≤ 0.01) in comparison to Caco-2 cells treated with control-GC-CM. In contrast, the mRNA expression levels of these three TJ proteins with strengthening activity remained unchanged or reduced following the ECOR12-GC-CM treatment. Notably, the incubation of Caco-2 cells with ECOR12-GM-CM resulted in the upregulation the leaky-gut-associated protein claudin-2 (*p* ≤ 0.05) (Figure 4). These results were consistent with the differential regulation of the TFF3 expression in LS174T cells by EcN and ECOR12 EVs. A positive correlation between highly secreted TFF3 levels and an increased expression of sealing TJ proteins was only achieved by the action of EcN EVs on intestinal goblet-like LS174T cells.

### 3.5. Immunofluorescence Microscopy Analysis of ZO-1 in Caco-2 Cells Treated with Conditioned Media from EV-Stimulated LS174T Cells

**Figure 5:**
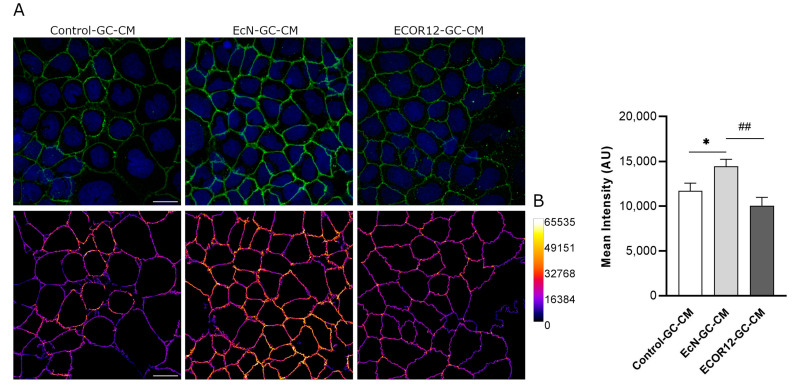
Immunofluorescence staining for ZO-1 in Caco-2 cells treated for 24 h with EV-stimulated LS174T conditioned media. (**A**) Representative confocal maximal projection images of cells treated with Control-GC-CM (left), EcN-GC-CM (middle), and ECOR12-GC-CM (right). Top row shows ZO-1 (green) and nuclei (blue) staining. Bottom row shows ZO-1 staining in Fire LUT after image processing. Calibration bar of Fire LUT intensity is shown on the right. Scale bar: 20 µm. (**B**) Quantification of the ZO-1 mean intensity in Caco-2 cells after the indicated treatments. Data are presented as mean ± SEM of arbitrary intensity units (AU) (n = 3 independent biological replicates). Statistical differences were assessed with one-way ANOVA, followed by post hoc Tukey’s. * *p* ≤ 0.05 vs. control group, and *## p* < 0.010 between EcN-GC-CM and ECOR12-GC-CM treatments.

In addition to the transcriptional regulation of the TJ proteins, TFF3 promotes a redistribution of ZO-1 from the cytoplasm to intercellular junctions [24,25]. To confirm the impact of EcN-GC-CM and ECOR12-GC-CM on the TJs, we carried out immunofluorescence staining, followed by confocal laser scanning microscopy of the ZO-1 in Caco-2 cell monolayers challenged with conditioned media collected from untreated or vesicle-treated LS174T cells. After 24 h of incubation, the cells were fixed and stained for ZO-1. Representative images are presented in Figure 5A. The upregulation of ZO-1 by EcN-GC-CM was correlated with an increased signal in the cell boundaries of the Caco-2 monolayers. Moreover, the ZO-1 signal was quantified in the TJs, as described in the Materials and Methods section (data collected from three independent biological experiments with five different fields of view with 30–40 cells each). Consistent with the TFF3 levels in LS174T conditioned media, the results revealed a statistically significant increase in the ZO-1 signal associated with the TJs in the cell boundaries of the Caco-2 monolayers treated with EcN-GC-CM, but not those treated with ECOR12-GC-CM (Figure 5B). 

### 3.6. Effect of the Conditioned Media from EV-Stimulated LS174T Cells on Wound Healing in Caco-2 Cells

**Figure 6:**
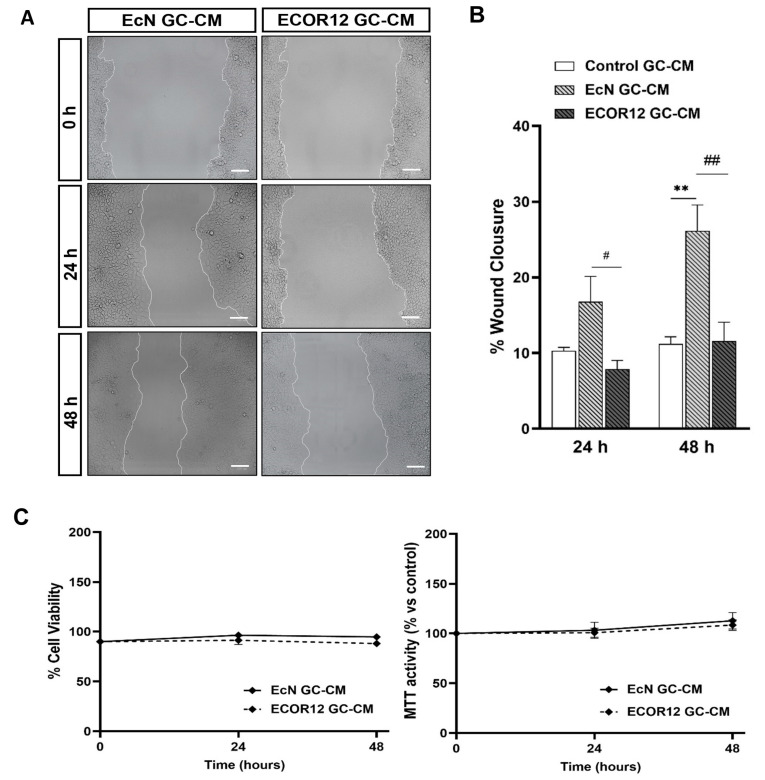
Conditioned media from LS174T cells treated with EcN EVs accelerates the wound closure in Caco-2 cells. Wounded confluent Caco-2 cell monolayers were incubated for 48 h with GC-CM (mixed with FBS-free RPMI at a ratio 1:1) collected from LS174T cells following stimulation with EcN EVs, ECOR12 EVs, or without any stimuli. Images of wound closure were taken at 0, 24, and 48 h of incubation. (**A**). Representative images of scratched areas at 0, 24, and 48 h are shown (Scale bar = 100 µm). (**B**) Analysis of wound closure. ImageJ was used to measure the width along the cell-free gap. Results are presented as the percentage of wound closure at 24 and 48 h compared to the wound at time 0 h and expressed as mean ± SEM. (**C**) Cell viability under the experimental conditions assessed with the MTT and trypan blue exclusion assays. In all panels, data are expressed as mean ± SEM from three independent experiments. Differences were evaluated with one-way ANOVA, followed by post hoc Tukey’s. ** *p* ≤ 0.01 vs. control group, and # *p* ≤ 0.05, ## *p* ≤ 0.01 between EcN-GC-CM and ECOR12-GC-CM treatments.

Since TFF3 plays a main role in tissue repair, the stimulatory effect of EcN EVs on the TFF3 secretion of goblet cells was further examined using the wound-healing assay in Caco-2 cells, as described in the Material and Methods section. In the absence of FBS, the conditioned media from LS174T cells stimulated with EcN EVs (EcN-GC-CM) significantly promoted the wound closure of the scratched Caco-2 cell monolayers when compared to the control treatment (control-GC-CM). For EcN-GC-CM, the percentage of lesion closure was 16.7% at 24 h and 26.12% (*p* ≤ 0.01) at 48 h compared to the wound at time 0, whereas for the control GC-CM, these values were about 14% at both 24 and 48 h. In contrast, ECOR12-GC-CM did not promote lesion repair (Figure 6A,B). The differential wound repair activity of EcN-GC-CM and ECOR12-GC-CM correlated with the TFF3 mRNA and protein levels expressed by LS174T cells in response to EcN or ECOR12 EVs. We also assessed the effect of the conditioned media on the cell viability. To this end, Caco-2 monolayers were incubated with the conditioned media (GC-CM:FBS-free RPMI; 1:1) under the same conditions of the wound healing and RT-qPCR assays, and the cell viability was evaluated at 24 and 48 h with the trypan blue exclusion and MTT tests (Figure 6C). The results showed that none of the GC-CM assayed was cytotoxic for Caco-2 cells under the experimental conditions. 

**Figure 7:**
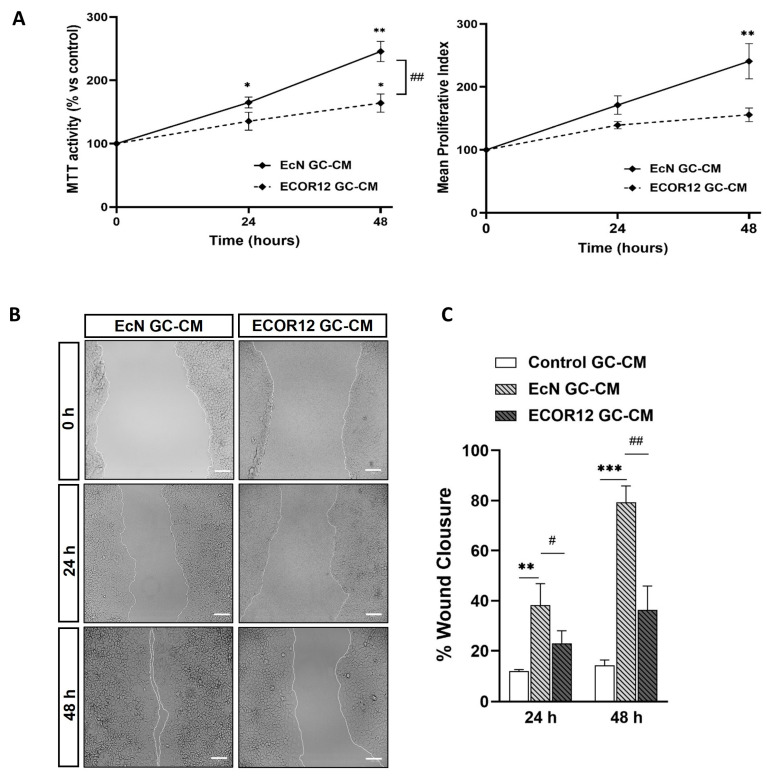
Conditioned media from LS174T cells treated with EcN EVs or ECOR12 EVs induce cell growth and improve wound repair in Caco-2 cells. Confluent Caco-2 cell monolayers were incubated for 48 h with GC-CM (mixed with 10% FBS-RPMI at a ratio 1:1 to reach 5% FBS final concentration) collected from LS174T cells following stimulation with EcN EVs, ECOR12 EVs, or without any stimuli. The effects on cell growth and wound healing were evaluated at 24 and 48 h incubation. (**A**) Cell growth was assessed with the MTT and trypan blue exclusion assays and the Mean Proliferative Index (MPI) was calculated as described in Methods. (**B**) Representative images of scratched areas at 0, 24, and 48 h are shown (Scale bar = 100 µm). (**C**) Analysis of wound closure. ImageJ was used to measure the width along the cell-free gap. Results are presented as the percentage of wound closure at 24 and 48 h compared to the wound at time 0 h. In all panels, data are expressed as mean ± SEM from three independent experiments. Differences were evaluated with one-way ANOVA, followed by post hoc Tukey’s. * *p* ≤ 0.05, ** *p* ≤ 0.01, *** *p* ≤ 0.001 vs. control group, and # *p* ≤ 0.05, ## *p* < 0.01 between EcN-GC-CM and ECOR12-GC-CM treatments.

To evaluate the effects of the GC-CM on Caco-2 cell proliferation, the conditioned media collected from the control and vesicle-stimulated LS174T cells were mixed with RPMI to reach a final FBS concentration of 5%. The cell proliferation was evaluated using the MTT assay at 24 and 48 h. The conditioned media collected from LS174T cells challenged with EcN EVs significantly stimulated the Caco-2 cell proliferation compared to both control-GC-CM and ECOR12-GC-CM (Figure 7A). 

The MPI values indicated that the highest proliferative activity was associated with EcN-GC-CM (Figure 7A, right panel). Consistently, in the scratch assay, EcN-GC-CM significantly enhanced wound closure compared to the control, reaching percentages of around 38% wound closure at 24 h (*p* ≤ 0.01) and over 79% at 48 h (*p* ≤ 0.001) (Figure 7B,C). The treatment with ECOR12-GC-CM also stimulated wound closure compared to the control, although the differences were not statistically significant. These results indicated that EcN EVs stimulate goblet cells to secrete factors that promote the cell proliferation of IECs.

## 4. Discussion

The lifestyle of modern societies has a great impact on human health. Stress, social habits, the Western diet, physical inactivity, and aging are the main factors that predispose people to a wide range of inflammatory (irritable bowel syndrome and IBD), metabolic (obesity, metabolic syndrome, chronic liver disease, and type 2 diabetes), and mental diseases (depression, anxiety, and behaviour disorders). Imbalances in the gut microbiota composition and diversity that result in increased intestinal permeability are common traits in all these diseases. The disruption of the epithelial barrier allows for the translocation of intestinal microbes and microbial components into the lamina propria. Translocated bacterial MAMPs activate the host immune system, triggering inflammatory responses that cause intestinal dysfunction. In addition, high levels of circulating pro-inflammatory mediators and microbial products negatively impact the normal communication networks between the gut and its microbiota with distal organs [2].

Importantly, there is currently no effective treatment available for IBD and chronic liver diseases [44,45]. The strong association between the gut microbiota and human health encourages the design of new microbiome-oriented nutritional and therapeutic strategies for restoring dysbiosis and host homeostasis. Diet is a main factor influencing the gut microbiota composition. Therefore, dietary strategies for manipulating the microbiota diversity are the focus of many studies. However, short-term interventions do not cause permanent microbiota changes and long-term interventions greatly depend on duration and dietary compliance [46]. In this context, probiotics are gaining increasing interest as a treatment option for gut microbiota manipulation and their impact on leaky-gut-associated disorders. However, the potential risk for immunocompromised individuals and the technological limitations to controlling bacterial viability are seen as potential limitations for their application as functional food products. 

Now, the interest in biotic well-being therapies is moving towards postbiotics [47,48]. The term postbiotic encompasses products derived from probiotics or good, beneficial bacteria that are generated during the fermentation process. These include bacterial cell components, biomolecules, metabolites, and secreted bioactive compounds. Postbiotics usually show good absorption, distribution, and a high capacity for signalling different organs and tissues to trigger specific host responses [5,49]. In this context, probiotic and microbiota EVs display the features to be considered as postbiotics, as they serve as vehicles for the delivery of functional bacterial molecules to distant cells and are important players in microbiota–host communication. Due to their nano-size structure, stability, and great variety of cargo molecules, microbiota-derived EVs can penetrate the mucus layer and modulate intestinal immune and defence responses. In addition, EVs from gut microbes can cross the epithelial barrier, enter the bloodstream, and reach the liver and distal tissues. 

Several in vivo studies in mouse models of IBD have shown that the oral administration of EVs from the probiotic EcN and *Clostridium butyricum* [17,50], or beneficial gut microbiota such as *Bacteroides fragilis* [51] or *Akkermansia muciniphila* [52], improve the intestinal barrier function through several mechanisms that include the modulation of inflammatory and immune responses, TJs, and mucin-related proteins. The efficacy of *A. muciniphila* EVs in the modulation of gut permeability has also been proven in a mouse model of diabetes induced by a high-fat diet [53]

IBD is a group of intestinal disorders, namely ulcerative colitis and Crohn’s disease, characterized by chronic inflammation. This condition results from a complex combination of factors, including the genetic traits of susceptible patients, disruption of the intestinal barrier, aberrant immune responses to luminal microbes and antigens, and dysbiosis. The dysfunction of the intestinal epithelial barrier is a key event in the development of IBD. The disruption of epithelial TJs and impaired mucosal repair exacerbate inflammatory responses and colitis. In the intestine, TFF3 secreted by goblet cells has a key role in the maintenance and repair of the intestinal mucosa barrier under physiological and pathophysiological conditions [23]. TFF3 is upregulated in response to intestinal mucosal damage to help cell migration for mucosa healing and decrease the epithelial permeability by regulating TJ proteins. However, the proinflammatory cytokines, IL-1β, IL-6, and TNF-α, cause the downregulation of TFF3, a mechanism that could contribute to ulceration and reductions in wound healing in IBD [54,55]. Moreover, the miRNAs dysregulated in IBD, such as miR-7-5p, may contribute to TFF3 downregulation at the posttranscriptional level [33]. 

Microbiota-derived EVs are known to modulate numerous functions that are essential to maintaining intestinal homeostasis in humans, including gut immunity and mucosal barrier function. While the effects of microbiota EVs on the regulation of epithelial tight junctions and immune responses is well documented [9], their role in TFF3 regulation is less studied. In this context, we previously showed that EVs from the probiotic EcN were able to counteract the reduced expression of TFF3 in an experimental model of DSS-induced colitis in mice [17].

Here, we investigate the molecular mechanisms activated by the EcN EVs responsible for TFF3 activation in goblet cells and their subsequent effects on intestinal epithelial barrier integrity and repair. To decipher whether these effects were strain specific, EVs from the commensal ECOR12 were included in this study. In this context, we have previously shown that EVs from the probiotic EcN and EVs from the commensal ECOR12 have different immunomodulatory [15,16] and barrier-strengthening activities. In in vitro models of intestinal epithelial cells (Caco-2 and T-84 cells), EcN EVs reinforced TJs by upregulating the expression of the main TJ proteins at the transcriptional level and the subcellular location of ZO1 associated with the cell membrane, whereas ECOR12 EVs did not regulate the TJ proteins or strengthen the epithelial cell barrier [13,14].

Concerning the effects on the TFF3 expression in the goblet cell line LS174T, the results from this study also showed a differential regulation of the TFF3 secretion by EcN and ECOR12 EVs. Only EVs from the probiotic EcN stimulated TFF3 production. Importantly, the TFF3 levels correlated with the differential regulation of the main regulators of the TFF3 expression, specifically TLR2 and miR-7-5p. It is known that the gut microbiota activates TFF3 expression through TLR2 signalling [29]. Our results indicated that EcN EVs stimulated the TFF3 production in goblet cells by upregulating TLR2. The involvement of the TLR2 signalling pathway was confirmed by using C29, a TLR2 inhibitor that binds to the intracellular TLR2-TIR domain [56]. Consistently, the activation of TFF3 expression and its secretion by EcN EVs was impaired in the cells incubated in the presence of C29. The inability of the ECOR12 EVs to activate TLR2 in the goblet cells may explain why the EVs from this commensal did not stimulate TFF3 production. 

As stated above, TFF3 levels can be downregulated at the posttranscriptional level by mir-7-5p. This microRNA binds to specific sequences at the 3′-UTR of TFF3 mRNA, promoting its degradation [33]. There is evidence that the activation of TFF3 activates the PI3K/AKt pathway, leading to the downregulation of miR-7-5p. Under healthy conditions, high expression levels of TFF3 correlate with a reduced miR-7-5p expression. In contrast, when miR-7-5p is upregulated (in IBD for instance), the degradation of TFF3 mRNA results in the inhibition of the PI3K/AKT pathway [26,27]. Notably, this pathway mediates the TFF3 effects on cell migration and mucosal repair [32,33]. To our knowledge, the regulation of miR-7-5p in goblet cells by microbiota EVs has not been explored yet. Our results confirm that the upregulation of TFF3 by EcN EVs correlates with reduced miR-7-5p levels, and that the inhibition of the PI3K pathway by LY294002 causes the upregulation of this microRNA and, thus, the downregulation of TFF3. Overall, these results suggest that the downregulation of miR-7-5p by EcN EVs may be indirect, through increased TFF3 production. On the contrary, ECOR12 EVs triggered the upregulation of miR-7-5p using a mechanism that did not involve the PI3K pathway. 

Since EcN EVs stimulated TFF3 production in the goblet cells, we hypothesized that EVs from this probiotic might indirectly reinforce epithelial TJs and promote mucosal restitution after epithelial injury. To prove these activities, as a model, we used Caco-2 cells incubated with the conditioned media collected from LS174T cells stimulated with the EcN or ECOR12 EVs (GC-CMs). Concerning the strengthening activity, the transcriptional regulation of ZO-1, occludin, and claudin-1 correlated with the TFF3 levels in the conditioned media. Only EcN-GC-CM upregulated these TJ proteins and promoted a redistribution of ZO-1 to the cell membrane. The indirect upregulation of these TJ proteins by goblet-cell-derived TFF3 has also been described for natural compounds with antioxidant activity [38]. Thus, besides the direct regulation of epithelial TJs [13,14], EcN EVs also reinforce the epithelial barrier indirectly through the activation of TFF3 in goblet cells. In contrast, no direct [14] or indirect strengthening effects are associated with ECOR12 EVs. Regarding barrier repair effects, a key step following injury is the migration of epithelial cells to the damaged area to reconstruct the cell monolayer, a process known to be stimulated by TFF3. In agreement with the upregulation of TFF3 by EcN EVs in LS174T cells, the derived conditioned media (EcN-GC-CM) significantly increased the wound closure of scratched Caco-2 cell monolayers. In the absence of FBS, this healing activity may be attributed to the TFF3 effects on epithelial cell migration. This fact is consistent with the lack of wound-healing activity of ECOR12-GC-CM, which displays TFF3 levels similar to that of control-GC-CM. In addition, EcN-GC-CM also stimulates Caco-2 cell proliferation in the presence of FBS and improves wound repair. Under these conditions, the positive effects of EcN-GC-CM on both cell migration and proliferation may explain the high percentage of wound closure values at 48 h (more than 4-fold) compared to the control-GC-CM. Although some reports do not attribute TFF3 to any activity in regulating epithelial cell proliferation [57,58], other studies have described that TFF3 can enhance cell proliferation [32,59]. The contradictory reports and divergences on the role of TFF3 in promoting cell proliferation may be attributed to differences in the cell lines and experimental conditions used. Whether the cell proliferative effects of EcN-GC-CM are mediated by factors released by goblet cells in response to microbiota EVs other than TFF3 cannot be excluded. In this sense, ECOR12-GC-CM tends to stimulate Caco-2 cell proliferation, but at a much lower extent. The fact that other factors could be upregulated in goblet cells by microbiota EVs is supported by the results from the wound-healing assays performed in LS174T, showing that ECOR12 EVs promote cell restitution without increasing TFF3 levels (Figure 3). In addition to TFF3, it is known that growth factors such as FGF1, FGF2, and VEGF-C secreted by goblet cells are important regulators of goblet cell restitution [60]. 

This study provides scientific knowledge on the differential regulation of TFF3 by microbiota EVs and the subsequent impact on barrier function and repair. Due to the important role of TFF3 in the mucosal protection of healthy intestinal tissue and the regeneration of its integrity after injury, this peptide is recognized as a potential therapeutic target in IBD. The scientific community is gradually moving towards personalized medicine, and the microbiome is an important target to consider in the future of medicine and nutritional approaches. Basic science to establish the molecular mechanisms elicited by microbiota/probiotic-specific active compounds, including derived EVs, is an important and necessary step before applying postbiotics as nutritional supplements in human healthcare. In the context of IBD, various treatment options have been developed to modify the pathogenesis factors involved in the disease progression [45]. In this sense, functional foods that improve intestinal health by promoting the repair and strengthening of the epithelial barrier could be used as therapeutic agents. Here, we show that EVs from the probiotic EcN promote these beneficial effects.

## 5. Conclusions

Novel pharmabiotics or nutritional formulations based on postbiotics, including EVs isolated from certain gut-beneficial microbes, could provide health benefits and avoid the potential risks of live bacteria administration. The results from this study contribute to understanding the molecular actors (microbiota EVs) connecting gut microbes to health. They may help in designing better interventions based on the use of specific microbiota bioactive compounds.

## Data Availability

The datasets generated and/or analyzed during the current study are available from the corresponding authors on reasonable request.

## Supplementary Materials

The following supporting information can be downloaded at: https://www.mdpi.com/article/10.3390/nu15112437/s1, Figure S1: Cell viability of LS174T cells at 6 h incubation with different concentrations of the indicated inhibitors ranging between 5 and 50 µM. Figure S2: MTT activity of LS174T cells at 24 h and 48 h incubation with EcN and ECOR12 EVs in RPMI containing 5% FBS.
