# Supplementary material for: Modulation of the Intestinal Barrier Integrity and Repair by Microbiota Extracellular Vesicles through the Differential Regulation of Trefoil Factor 3 in LS174T Goblet Cells
Source: Nutrients. 2023 May 24;15(11):2437. doi: 10.3390/nu15112437 (PMC10255446; doi:10.3390/nu15112437)
Supplement: Supplementary file 1 [file nutrients-15-02437-s001.zip › nutrients-2395808-supplementary.pdf]

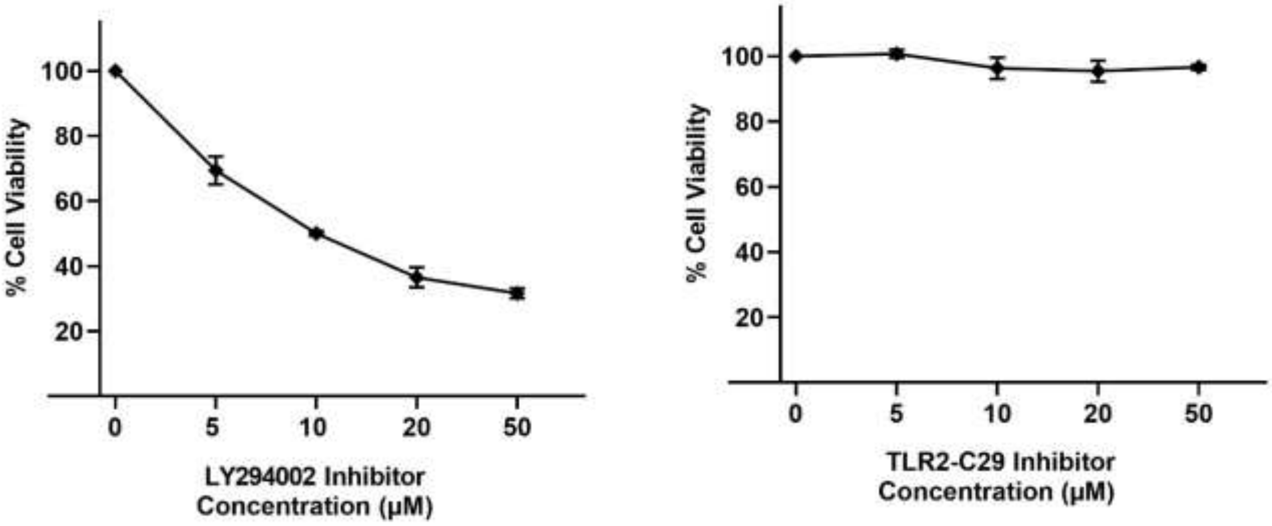

Supplementary Figure S1. Cell viability of LS174T cells at 6 h incubation with different concentrations of the indicated inhibitors ranging between 5 and 50 μM. Cell viability was assayed by the MTT reduction method and expressed as percentage of untreated control cells. Values represent the Mean ± SEM of three independent replicates.

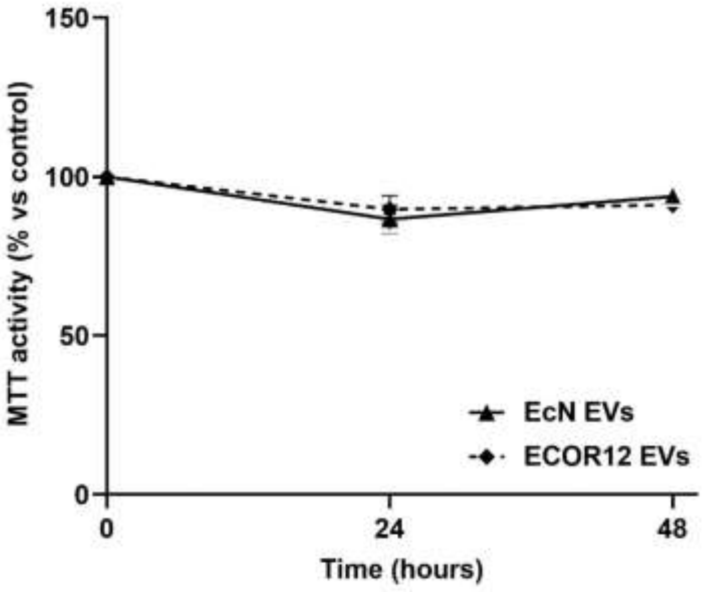

Supplementary Figure S2. MTT activity of LS174T cells at 24 h and 48 h incubation with EcN and ECOR12 EVs in RPMI containing 5% FBS. Data are expressed as percentage of untreated control cells. Values represent the Mean ± SEM of three independent replicates.
